# A Rasch analysis of the self-administered Foot Health Assessment Instrument (S-FHAI)

**DOI:** 10.1186/s12912-021-00625-z

**Published:** 2021-06-15

**Authors:** Minna Stolt, Anders Kottorp, Riitta Suhonen

**Affiliations:** 1 Department of Nursing Science, University of Turku, 20014 Turku, Finland; 2 Faculty of Health and Society, Malmö University, Malmö, Sweden; 3 Turku University Hospital and City of Turku Welfare Division, Turku, Finland

**Keywords:** Foot health, Psychometric testing, Rasch analysis, Self-assessment

## Abstract

**Background:**

Reliable and valid measurement is the foundation of evidence-based practice. The self-administered Foot Health Assessment Instrument (S-FHAI) was recently developed to measure patients’ evaluations of their own foot health. Evidence regarding the psychometric properties of the S-FHAI is limited. The aim of this study was to investigate those properties by using a Rasch analysis.

**Methods:**

This methodological study analysed secondary data that was collected from nurses (*n* = 411) in 2015. The psychometric properties of the S-FHAI were evaluated using the Rasch model. Unidimensionality was analysed first, followed by item functioning, person misfit and differential item functioning (DIF).

**Results:**

The S-FHAI demonstrated evidence of unidimensionality, with an acceptable item fit according to the Rasch model. Person fit and person separation were low, however, indicating restricted separation among different respondents. Item separation was high, demonstrating clear discrimination between the items. No DIF was detected in relation to gender, but significant DIF was demonstrated in relation to age for 6 of the 25 items.

**Conclusions:**

The S-FHAI has potential for use in investigating self-reported foot health. The Rasch analysis revealed that the psychometric properties of the instrument were acceptable, although some issues should be addressed to improve the scale. In future, it may be beneficial to analyse the sensitivity of the items and to test the S-FHAI in more diverse patient populations.

## Background

Foot health is part of general health. Healthy feet help make pain-free activity possible for people in every age group. This is especially important in occupations that involve long periods of standing or walking, such as nursing. The prevalence of foot problems among nurses is high [1, 2]. Nurses may experience foot problems that stem from long-term disease (e.g. diabetes, rheumatoid arthritis) or a limited ability in foot self-care, and these issues can affect their ability to work [1–4]. Foot problems are more prevalent among female and associated with lower quality of life [5, 6]. The importance of foot health is usually noticed when problems causes pain or discomfort. However, preventative monitoring of foot health is seldom conducted [7]. Therefore, the assessment of foot problems using suitable and reliable instruments is important to identify the problems and promote foot health.

Several instruments are available for measuring foot health. These tools have different aims and perspectives, and they may be designed for either patients or podiatrists to use. Most of the instruments available are related to a specific disease or foot problem; for example, the Manchester Foot Pain and Disability Index [8] measures pain specifically related to a foot disability. Others measure foot-related outcomes on a more general level, such as quality of life related to foot health (e.g. the Foot Health Status Questionnaire) [9] or functional limitation (e.g. the Foot Impact Scale – Dutch version) [10]. The Foot Health Assessment Instrument (FHAI) [11]; is an objective tool used by nurses to measure foot health in older people. Many of the instruments focusing on foot health are administered predominantly by podiatrists, with few considering the patient’s perspective. To respond to this need, a self-administered version of the Foot Health Assessment Instrument was developed to measure foot health as reported by patients.

Testing the psychometric properties of foot health instruments is continuous process. Psychometric properties of the instrument should be evaluated every time when the instrument is used in different sample, setting or culture [12]. There are several different language versions of previously validated foot health instruments, for example, the Spanish Bristol Foot Score [13], the Spanish ROWAN Foot Pain Assessment Questionnaire [14] and Turkish version of the Foot Function Index [15]. However, the evaluation of psychometric properties of foot health instruments is largely focused on using classical test theory approach.

The Rasch analysis is a modern test theory approach to assessing the psychometric properties of instruments. It can be used either when developing new instruments or when evaluating and revising existing ones [16]. The Rasch analysis examines the items included in the instrument and the people who are using it. The main assumption in a Rasch analysis is that the probability of responding correctly or incorrectly to a single item is related to both the person’s ability and the difficulty of the item [17]. The Rasch analysis provides a wide range of evidence of internal construct validity, such as unidimensionality, category function, item and person separation, and differential item functioning (DIF) [18].

In foot health research, the Rasch analysis has been applied to detect items that are misfits in an instrument and to evaluate its psychometric properties with national and international samples. For example, in the process of developing the Foot Posture Index, the Rasch analysis was used to quantify variation in the position of the foot, detect problematic items and provide evidence of internal construct validity [19]. When the Foot Impact Scale (which measures foot-related impairment and disability) was translated into Dutch, a Rasch analysis was used to evaluate internal construct validity, which led to the deletion of two items [10]. After these deletions, the Foot Impact Scale – Dutch version demonstrated unidimensionality and acceptable goodness-of-fit values [10]. In the context of cross-cultural validation, a Rasch analysis was used to confirm the validity and reliability of the Foot and Ankle Ability Measure [20] and the Manchester Foot Pain and Disability Index – Spanish version [21]. When applied to the American Orthopaedic Foot and Ankle Society Score [22] in a sample of Brazilian patients with rheumatoid arthritis, the Rasch analysis demonstrated one item that did not fit, thus supporting the refinement of the tool.

The development and testing of instruments is an ongoing process. The aim of this study, therefore, was to understand more about the psychometric properties of the S-FHAI by applying a Rasch analysis to secondary data. Five stepwise research questions were set:

1. What is the rating scale functioning of the S-FHAI?
2. Do the items in the S-FHAI support a unidimensional underlying construct? In particular:

- (a) Do the patterns in the participants’ responses to the items demonstrate acceptable goodness-of-fit with the Rasch model?
- (b) Is most of the variance explained by a single underlying construct?

(3) Do the individual responses match the responses expected according to the Rasch model?
(4) Does the S-FHAI separate the sample into a sufficient number of distinct levels of foot health?
(5) Are the item difficulty calibrations stable in relation to gender and age?

The goal was to provide information about the validity and reliability of the S-FHAI and to support its use in clinical practice.

## Method

The methodological design used in this study involved the analysis of secondary data (*n* = 411) from a previously reported study [3]. The data were collected electronically. A random sample was taken from two national associations in Finland: the Finnish Nurses’ Association, which represents registered nurses, and the Finnish Union of Practical Nurses, which represents licensed practical nurses. The minimum sample size was estimated with the rule of thumb: 10 x number of items + drop out = sample size [23]: 10 × 25 + 50 = 300 in this study.

### Instrument

The S-FHAI measures a person’s current level of foot health. It consists of 25 items divided into four subcategories: skin health (11 items), nail health (4 items), foot structure (5 items) and foot pain (5 items, focusing on intensity and location). The response options in the first three subcategories are dichotomous (yes or no), and in the fourth subcategory (foot pain) a four-point Likert scale (0 to 4) is used. The S-FHAI is based on the FHAI [11], which was developed and tested for use by nurses to assess foot health in older people. To improve its use in cross-sectional studies, a self-administered version was constructed [3]. Three items related to the objective assessment of foot sensation and arterial blood supply (palpation of arteria dorsalis pedis and tibialis pulses) were omitted from the S-FHAI. These were replaced with the following five items: blisters, cold feet, muscle cramps, foot sweating and sensation of burning feet). These modifications were discussed and agreed with experts in the fields of podiatry and nursing, and were guided by the previous literature [e.g. 1]. The S-FHAI has been used successfully in Finnish studies measuring nurses’ foot health [3, 4]. Both Finnish and English versions of the S-FHAI exist, however this study focus on the Finnish version.

### Analysis

A Rasch partial credit model application was used to assess unidimensionality, item fit, person fit, person separation and item hierarchy. A summary of the steps taken in this approach is provided in Table 1, drawing upon examples from Bonsaksen and colleagues [24] and Lerdal and colleagues [25]. In this study, the data were analysed using WINSTEPS software (Version 4.4.8) [26].

**Table 1:** Rasch approach to analysing the S-FHAI

Step	Psychometric property	Statistical approach and criteria	Results (original S-FHAI)
1	***Rating scale functioning***: Does the rating scale function consistently across the items?	• Average measures for each category and threshold on each item should advance monotonically • Zstd values < 2.0 in outfit mean square (MnSq) values for step category calibrations [27]	Four items had less than 10 responses per category. Two categories were combined, and then only two items did not meet the criteria
2	***Internal scale validity***: How closely do the item responses match the responses expected according to the Rasch model?	• Item goodness-of-fit values with MnSq values between 0.6 and 1.4 [28]	All items met the criterion
3	***Internal scale validity***: Is the scale unidimensional?	• Principal component analysis, with the first component explaining ≥ 50 % of total variance and any additional component explaining < 5 % (or eigenvalue < 2.0) of the remaining variance [26]	First component explained 54.2 % of total variance, and second component explained 5.9 % (eigenvalue 3.25) of total variance
4	***Person-response validity***: How closely do the individual responses match the responses expected according to the Rasch model?	• Person goodness-of-fit statistics with infit MnSq < 1.4 and Zsrd value ≤ 2.0 [29] • ≤ 5 % of the sample fails to demonstrate acceptable goodness-of-fit values [29]	34 % of participants failed to demonstrate acceptable goodness-of-fit values
5	***Person-separation reliability***: Can the S-FHAI distinguish between two distinct foot-health groups in the sample?	• Person-separation index ≥ 2.0 [30]	0.37
6	***Differential test functioning (DIF)***: Are the item difficulty calibrations stable in relation to gender and age?	• Mantel–Haenszel statistic for polytomous scales using log-odds estimators in WINSTEPS software (P < 0.01) [31, 32]	Gender: all item difficulty calibrations were stable Age: six items had DIF in relation to age

To determine whether the number of response options was appropriate, the rating scale functioning was examined. This included assessing category frequencies, average measures, infit and outfit mean squares and threshold calibrations. For category frequencies, the minimum requirement is 10 responses per category and the average measures should increase monotonically [33]. If the rating scale demonstrates a low number of category frequencies or disordered average measures, it may be appropriate to combine some of the response categories [33].

Unidimensionality is a main requirement of the Rasch model. For an instrument to be unidimensional, all the items must measure a single construct [17]. Unidimensionality can be assessed by looking at the fit statistics for the items and carrying out a principal components analysis (PCA) of the residuals [34]. Fit statistics are used to identify the items or participants whose responses deviated from what was expected. The fit statistics are normalized mean square residuals and are reported in two ways: infit and outfit statistics. Infit statistics are sensitive to unexpected responses close to an item’s measure, and outfit statistics are sensitive to unexpected responses far from an item’s measure [17]. In addition, both the infit and outfit statistics have two forms: mean square (MnSq) and standardized mean square (Zstd) [17]. A MnSq value greater than 1.4 or a Zstd value greater than 2.0 indicates a misfit, which means that the item’s performance does not match the expectations of the Rasch model. Infit values of less than 0.6 associated with a Zstd value of -2 suggest that an item is not contributing independent information [17, 34–36].

The internal scale validity of the S-FHAI was further examined using a principal component analysis of residuals. For the instrument to demonstrate unidimensionality, the first component should explain more than 50 % of the total variance and any other components should explain less than 5 % of the remaining variance [26]. Person fit provides evidence of person-response validity. This was evaluated by inspecting the person goodness-of-fit values. The criteria were set to ≤ 1.4 log-odds units (logits) and an associated Zstd value < 2 [37, 38].

Person reliability and person separation measure how the instrument distinguishes between respondents. The items must be sufficiently separated in terms of difficulty to identify the direction and meaning of the latent scale [39, 40]. To determine the extent to which the S-FHAI distinguishes among people with different levels of foot health, the separation index was calculated. The separation index criterion was set at ≥ 2.0, and reliability was set at ≥ 0.80. Item reliability was calculated in order to examine the degree to which the item response categories reflected increasing levels of difficulty (item separation with the criterion set at ≥ 2.0 and reliability set at ≥ 0.80). A separation index of 1.5 divides the respondents into two strata (high and low); an index of 2.0 three strata (high, moderate and low); and an index of 3.0 four levels of strata (high, above average, below average and low) [41, 42]. In classical test theory, person separation corresponds to reliability. In the Rasch analysis its meaning is not as pivotal; nevertheless, the index provides some insight into the power of the analysis of fit [41].

The hierarchy of the items determines the order of item difficulty in relation to the distribution of person ability. The Rasch model provides estimates of the item locations (calibrations) that define the order of the items along a measurement continuum [35]. The item calibration provides the hierarchical order of the severity or difficulty of the items on the scale. The Rasch model indicates how well the different items fit into a group of subjects [36]. Item calibration is described in log-odds units (logits), where a greater magnitude represents increasing item difficulty [35, 43]. Ideally, the distribution of the items matches the distribution of the participants [35, 43]. In this study, the item difficulty was analysed by using an item map and by visually evaluating where the items were located on the continuum.

DIF was used to assess whether or not the item difficulty calibrations were stable in relation to gender (male or female) and age. To analyse the DIF in relation to age, the sample was divided into two groups based on mean age: the age ranged from 19 to 44 in one group and from 45 to 65 in the other. The goal was to provide evidence of the internal structure of the S-FHAI and any potential for unfairness in testing [44].

### Ethical considerations

Good scientific practice [45] was followed throughout the research process. Ethical approval was obtained from the Ethics Committee at the University of Turku (code: 14/2015, 23.2.2015) and permission to conduct the study was sought in accordance with national guidelines. All of the participants received information in writing about the purpose of the study, the fact that taking part was voluntary, and the anonymity and confidentiality of the reporting. Written informed consent was obtained from each participant.

## Results

### Description of the participants

In total, responses from 411 nurses were included in the Rasch analysis. The mean age of the participants was 44 years (range 19–65, SD 11.6). Two-thirds of the participants were licensed practical nurses (*n* = 271, 66 %) and the rest were registered nurses (*n* = 140, 34 %). The participants had worked in health care for an average of 14 years (range 0.5–41, SD 10.3).

### Rating scale functioning

The rating scale functioning was assessed to check if the S-FHAI’s rating scale was being used as intended. In this dataset, four items (22, 23, 24 and 25) did not reach the target of 10 responses per category. Therefore, to obtain more stable estimates on item difficulty, the categories of 4 (strong pain) and 5 (worst imaginable pain) were combined in items 22–25, which measured incidences of pain in certain areas of the foot. After these modifications had been made, the average measures for each category and the thresholds advanced monotonically, except for two items (pain in the toes and pain in the sole). All the response options in all the items were used.

### Item fit

On the basis of item fit values, the item fit statistics were acceptable for all items and the loading was within the recommended range (Table 2). The item MnSq ranged from 1.14 to 0.91, and the Zstd values ranged from 1.33 to -2.68.

**Table 2:** S-FHAI item statistics in order of difficulty (from hardest to easiest)

				Infit		Outfit	
Item number	Abbreviated item	Measure	SE	MnSq	Zstd	MnSq	Zstd
22	Pain in the toes	2.01	0.07	1.14	1.17	1.93	4.47
25	Pain in the ankle	1.72	0.06	1.06	0.70	1.33	2.67
23	Pain in the sole of the foot	1.69	0.06	0.98	-0.20	1.14	1.22
6	Blisters	-2.41	0.23	1.00	0.09	1.13	0.59
24	Pain in the heel	1.81	0.06	0.92	-0.77	1.13	0.85
15	Fungal infection in the nail	-2.95	0.30	1.02	0.15	1.12	0.48
20	High arch	-1.83	0.18	1.05	0.38	1.11	0.67
9	Burning feet	-0.61	0.12	1.00	-0.03	1.07	0.85
5	Verruca	-2.08	0.20	1.00	0.04	1.06	0.35
21	Foot pain	1.41	0.11	1.05	1.33	1.04	0.86
16	Hallux valgus	-0.92	0.13	1.01	0.10	1.03	0.31
19	Low arch	-0.36	0.11	1.01	0.26	1.03	0.47
1	Maceration	3.04	0.17	0.99	-0.03	1.02	0.20
10	Cold feet	0.62	0.10	1.01	0.46	1.02	0.75
7	Oedema	0.28	0.10	0.99	-0.19	1.01	0.37
11	Muscle cramps	0.60	0.10	1.00	-0.16	1.01	0.36
8	Sweating	0.50	0.10	0.98	-0.68	0.98	-0.71
17	Taylor’s bunion	-1.40	0.16	0.98	-0.14	0.93	-0.53
18	Hammer toes	-1.79	0.18	0.98	-0.08	0.92	0.46
3	Fissures	0.41	0.10	0.96	-1.51	0.95	-1.55
12	Ingrown toenails	-1.36	0.15	0.96	-0.29	0.94	-0.43
2	Xerosis	1.98	0.12	0.94	-1.01	0.87	-1.66
4	Corns or callus	0.74	0.10	0.94	-2.68	0.93	-2.65
13	Thickened toenails	-0.30	0.11	0.91	-1.77	0.88	-2.11
13	Discoloured toenails	-0.80	0.13	0.91	-1.14	0.86	-1.60

*Infit MnSq* mean square standardized residuals; *SE* standard error; *Zstd* standardized Z-values

### Unidimensionality

The first component explained 54.2 % of the variance in the data, indicating that the S-FHAI had a satisfactory level of unidimensionality. The largest first contrast component explained 5.9 % (eigenvalue 3.25) of the variance, while the second largest explained 3.3 % (eigenvalue 1.79), which supported internal scale validity.

### Person fit

The majority of the participants had acceptable goodness-of-fit with Rasch model. However, a considerable number were misfits. The goodness-of-fit values from 62 participants (15 %) were above the criterion of 1.4, and values from 79 participants (19 %) below the criterion of 0.6. Therefore, 34 % of people (*n* = 141) did not demonstrate acceptable fit to the Rasch model.

### Separation

**Fig. 1:**
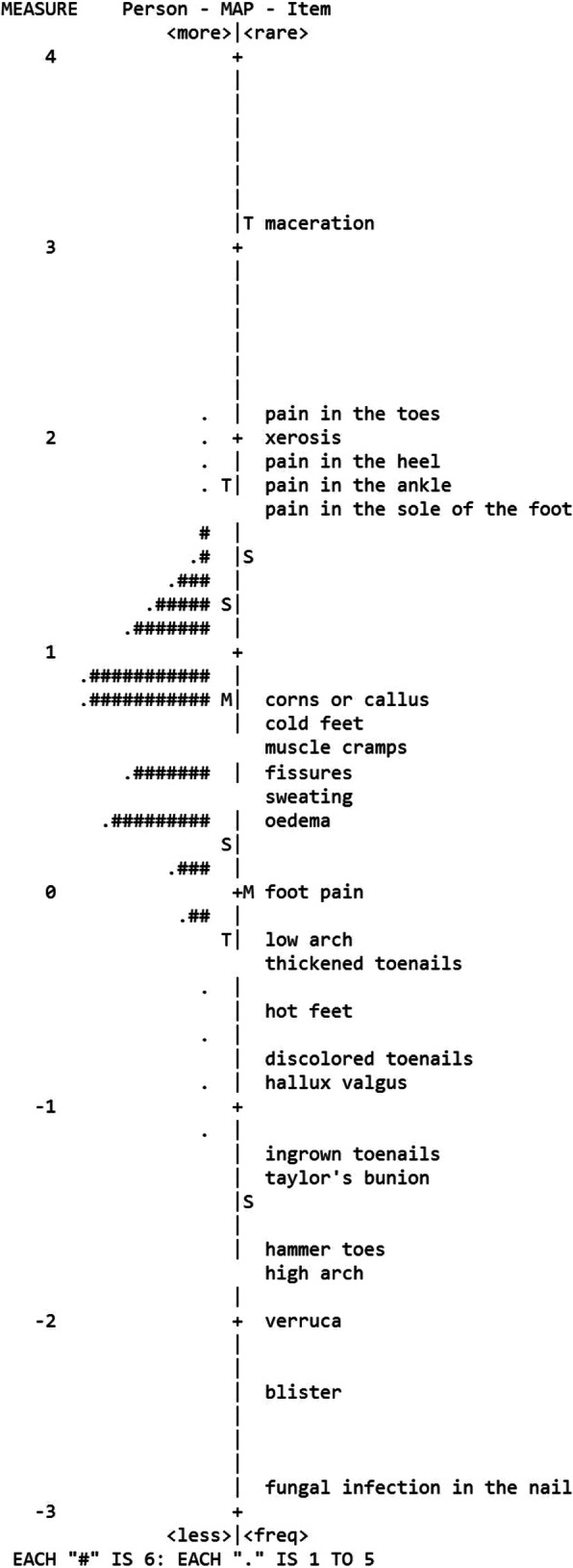
Item map (S-FHAI 25 items)

The figure for person separation was 0.37, which indicated that there was a restricted level of separation among the included participants. The figure for item separation was 10.95, which indicated that the instrument had a good ability to discriminate between and separate the items. The item hierarchy (item map) demonstrates how the items and participants fit together on a continuum. In this study, the participants tended to be located higher than the items. This indicates that the ability of this sample was higher than the ability reflected in the items. The mean of the item measures was less than 1 standard deviation lower than the mean of the person measures, which indicates that the test-item targeting is satisfactory (Fig. 1).

### Differential item functioning (DIF)

The presence of DIF was analysed in relation to gender and age. The analyses revealed that all 25 items in the S-FHAI functioned in a similar manner, regardless of gender. However, for age the DIF was significant for six items: fissures, sweating, cold feet, ingrown toenails, thickened toenails and hallux valgus. To check the presence of age-related DIF, the participants were grouped into two new age categories: those under 50 years and those 50 years or over. Even after making this modification, the age-related DIF remained evident in the same six items.

## Discussion

This study evaluated the psychometrics of the S-FHAI from the perspective of item response theory by using a Rasch analysis on responses from a sample of nurses. Mixed findings were demonstrated for the S-FHAI in this cohort. The instrument’s internal scale validity was supported in terms of unidimensionality (54.2 %) and all items had an acceptable fit. However, person fit and person separation were poor. In addition, there was significant DIF related to age.

Despite the good internal scale validity in this study, the person-response validity was limited. The percentage of participants demonstrating an unacceptable fit with the Rasch model was higher (34 %) than the criterion (< 5 %). When the goodness-of-fit statistics are higher than expected, this “person misfit” reflects a higher number of unexpected response patterns patterns from the respondents and casts doubt on inferences about a person’s score [46]. The S-FHAI was administered by the participants themselves, so it is important to view these findings in relation to the sample. On the basis of the person-item map, where lower scores on single items were not clearly associated with certain items but were more individually distributed, we can assume that the sample’s foot health was good or excellent overall,. This may explain the larger proportion of participants with higher goodness-of-fit statistics. A more in-depth analysis of a sample that demonstrated higher goodness-of-fit statistics could reveal if lower responses on specific items for specific subgroups could explain this pattern. A large proportion of participants with goodness-of-fit statistics that are lower than expected demonstrates “too expected” response patterns, which may be viewed as less of a problem from the perspective of validity.

In future evaluations of the S-FHAI, it will therefore be vital to analyse variables relating to the participants’ background when assessing item difficulty. The most difficult items (e.g. nail fungal infections) may be too complicated to identify for participants with a low level of education, as poor knowledge of foot-health issues – or guessing – could affect the responses on this test item. The participants in this study were nursing professionals, so they should have been able to recognize the relevant changes in their feet. However, if their feet were overall healthy, they might not have considered minor foot-health issues or have been able to identify them. Future studies of the S-FHAI in more healthy populations should therefore combine objective outcomes regarding foot health with the subjective perspective gathered with this tool. The objective assessment of foot health may then also potentially explain the unexpected responses among individuals resulting in a higher than expected level of misfit.

A low person separation (0.37) was evident in the S-FHAI, indicating threats related to sensitivity within this population. It is likely that this low level of separation was caused by the mismatch between the item difficulty and the participants. This may indicate that the S-FHAI can detect people with serious foot problems, but cannot differentiate between those with good or excellent foot health, as might have been the case in this generally “healthy” sample. Nevertheless, the poor separation limits the usefulness of the S-FHAI when it is applied in different populations. Although the findings suggest that the sensitivity of the S-FHAI may be limited when used in more generic and healthy samples, its sensitivity among people whose foot health is poor is still unknown. The S-FHAI is a subjective instrument that can be used to evaluate foot health as reported by patients, so it is important to further analyse whether the items have enough sensitivity to separate people with poorer foot health and also to detect changes over time among these people.

The separation capability of the tool could be improved by implementing several suggestions. First, the number of categories per item ranged from two to five. Therefore, person reliability could be improved by revising the response scale to include more options, such as “no problem”, “slight problem” and “severe problem”. Second, with only 25 items the S-FHAI is a relatively short scale. Therefore, person separation could be improved by lengthening the scale to include more items, especially the more “challenging” ones. These suggested revisions should be done in accordance with the theoretical framework behind the S-FHAI. Item separation was high (10.95) in this sample and supported the hierarchical way in which the items were organized in the S-FHAI [17]. The sample size was large (*n* = 411), which usually results in strong item reliability. It may also require a discussion about the aims of this self-assessment; is it to detect minor issues based on subjective responses in overall healthy samples, or is it to detect those that are in major need of interventions in order to improve foot health? Depending on the aims of the tool, the mismatch between an overall healthy sample and the items can be a serious limitation or not.

DIF was detected in relation to age for six items: fissures, sweating, cold feet, ingrown toenails, thickened toenails and hallux valgus. This is logical, as these foot-health problems are common in an aging population (e.g. [47, 48]). There was no DIF related to gender. To ensure comparable measures across different respondents, further analysis should be carried out on these six items to identify why the participants responded differently to those items.

The results of this study suggest that it would be beneficial to further revise, adapt and improve the cross-cultural validity of the S-FHAI scale, as it possesses some important qualities and has potential for investigating self-reported foot health. On the basis of the Rasch analysis, the psychometric properties of the S-FHAI were acceptable, in spite of some issues that should be addressed. In future, analysing the sensitivity of the items could be of benefit. The S-FHAI should also be tested in more diverse patient populations, especially those including people with lower levels of foot health, as it is more important to detect these from a health intervention perspective. The item hierarchy of the S-FHAI also informs which foot health items are relatively easy to subjectively detect and/or more frequently perceived within a population, to more challenging items that may be harder to detect and/or are less frequently perceived. This information may therefore also inform health professions to specific interventions.

### Methodological considerations

There were limitations in this study. The analysis relied on secondary data, which had been collected from one country. The sample may not be representative when compared with a larger population of nurses. However, the fact that the data were obtained from two national associations is a strength and makes it possible to carry out comprehensive statistical tests. Second, the data collected were the result of self-assessment. Self-assessment has been criticized for producing health-related results that are better than respondents’ real health status [49]. Despite this, the evaluations in this sample can be viewed as reasonable, as the participants had received nursing education and were therefore familiar with foot-related issues. The lack of an objective outcome in relation to foot health in this study limits the outcomes to support validity in relation to response processes and internal structure, but not validity evidence in relation to other variables [50]. Finally, the current findings can be generalized to this sample of nurses only and warrant exploration in other populations.

## Conclusions

In conclusion, the S-FHAI showed strong unidimensionality, and the goodness-of-fit values were satisfactory for all the items. The higher proportion of persons demonstrating misfit indicates the need for more detailed information about the foot health characteristics of this overall healthy sample, in order to detect if this a generic validity problem of the tool, or more sample-related. The low separation of the tool also requires further discussions about the aims and target groups for the S-FHAI. Future adaptation and cross-cultural validation analyses are needed to test the sensitivity and specificity of the S-FHAI in different samples; for example, using receiver operating characteristic curve (ROC) curves in relation to more objective outcomes.

## Data Availability

The datasets used and analysed during the current study are available from the corresponding author upon reasonable request.

## Abbreviations

DIF: Differential item functioning.
FHAI: The Foot Health Assessment Instrument.
logits: Log-odds units.
MnSq: Mean square.
PCA: Principal components analysis.
ROC: Receiver operating characteristic curve.
SD: Standard deviation.
SE: Standard error.
S-FHAI: The self-administered Foot Health Assessment Instrument.
Zstd: Standardized mean square.
